# Understanding Long COVID Among Young People in Victoria, Australia: Prevalence, Impact, and Associated Factors

**DOI:** 10.1002/puh2.70144

**Published:** 2025-10-21

**Authors:** Robin Engelberts, Caitlin H. Douglass, Ana Orozco, Sarah Eddy, Anna Lee Wilkinson, Aimée Altermatt, Suzanne van den Toren, Megan S. C. Lim

**Affiliations:** ^1^ Disease Elimination Burnet Institute Melbourne Victoria Australia; ^2^ Medical School Erasmus University Rotterdam, Erasmus Medical Center Rotterdam The Netherlands; ^3^ School of Population and Global Health University of Melbourne Carlton Victoria Australia; ^4^ School of Public Health and Preventative Medicine Monash University Melbourne Victoria Australia; ^5^ Department of Public Health Erasmus Medical Center Rotterdam The Netherlands; ^6^ Department of Health Albert Schweitzer Hospital Dordrecht The Netherlands

**Keywords:** Australia, COVID‐19, long COVID, young people

## Abstract

**Introduction:**

Long COVID is a significant public health concern. This study aimed to identify the prevalence, impact, and factors associated with long COVID among young people in Victoria, Australia.

**Methods:**

From April to June 2023, we conducted a cross‐sectional online survey of people aged 15–29 years. Participants reported if they had ever experienced long COVID (defined as COVID‐19 symptoms for more than 4 weeks) and the impact on their daily functioning. We used multivariable logistic regression to compare participants who reported long COVID with participants who reported acute COVID‐19.

**Results:**

Among 765 participants, 11.2% reported they had ever had long COVID; however, only 1 in 10 had been diagnosed by a medical practitioner. Compared to those without prolonged symptoms, participants reporting long COVID were younger (adjusted odds ratio [aOR]: 0.92; 95% confidence interval [CI]: 0.85–0.99), reported worsened general health (aOR: 8.22; 95% CI: 4.34–15.56), expressed greater concern about getting long COVID again (aOR: 1.20; 95% CI: 1.09–1.33), and had more family or friends who also experienced long COVID (aOR: 4.43; 95% CI: 1.92–10.19). In the past 4 weeks, 79.1% of participants with current long COVID reported difficulties with performing work, and 79.1% accomplished less than desired.

**Conclusions:**

One in 10 participants aged 15–29 years experienced long COVID, and most reported negative impacts on their daily life. General practitioners should be aware of the high burden of suspected long COVID in young people and consider supports to mitigate its effects on the health and well‐being of this population.

## Graphical abstract

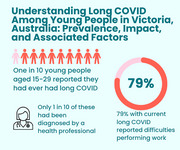

## 1 Introduction

Long COVID includes symptoms of fatigue or shortness of breath, persisting for either ≥4 [1] or ≥12 weeks [2] after initial infection, depending on the applied definition. As of August 2022, one study found that 4.7% of Australian adults (or approximately 10% of those who had experienced COVID‐19) had or were experiencing long COVID symptoms lasting 12 weeks or more [3]. This cohort study also found that these participants faced considerably greater difficulties with their daily activities compared to before getting COVID‐19 [3]. Most research on long COVID has focused on adults.

A small number of studies worldwide have investigated the prevalence of long COVID specifically in young people. For example, as of October 2023, an ongoing cohort study from the United States (US) reported that 16% of young people aged 18–29 years had ever experienced long COVID symptoms persisting for 12 weeks or more [4]. An ongoing cohort study from the United Kingdom (UK) showed that as of March 2023, 1.4% of young people aged 17–24 years had current self‐reported long COVID symptoms persisting for 12 weeks or more [5]. Up to now, there has been an insufficient description of the prevalence of long COVID among young people in Australia.

Emerging evidence has documented the serious impacts of long COVID on young people, including severe fatigue and difficulties navigating education, social activities, and the healthcare system [6]. A cohort study conducted in the UK involving approximately 13,000 young people showed that nearly half of those with long COVID felt they had fallen behind their classmates due to the pandemic [7].

Other studies have also explored the risk factors for long COVID in young people. Multiple potential biological risk factors for long COVID in young people have been identified, such as older age [8], severe acute COVID‐19 illness [8], and preexisting chronic conditions [9]. Limited research has explored the role of social determinants in the risk profile of long COVID. Understanding these social factors is crucial as some groups are disproportionally impacted by the condition. For example, a UK cohort study of nearly 13,000 young people showed that those from poorer areas were more likely to report symptoms of long COVID [7]. Moreover, a higher prevalence of long COVID has been reported in certain ethnicities in the US, including people of Hispanic or Latino heritage [4].

### 1.1 Aims

Research on the prevalence and impact of long COVID among young people in Australia could result in greater awareness of its effect on young people [10]. This may assist in providing care and support for young people experiencing long COVID symptoms. Better understanding of how social and behavioral factors influence long COVID risk could direct efforts toward healthcare equity. Our study aims to estimate the prevalence and impact of long COVID among young Victorians and identify differences in demographic and behavioral characteristics between young people with long COVID and those who had COVID‐19 without prolonged symptoms.

## 2 Methods

### 2.1 Study Design

This study used data from the Sex, Drugs, and Rock‘n'Roll 2023 survey [11]. This is an annual cross‐sectional survey conducted online since 2015. The survey included 61 questions on multiple health topics, including 12 questions on COVID‐19 and long COVID (see Table S1). The survey was hosted in Research Electronic Data Capture (REDCap) [12]. The survey took approximately 20 min to complete.

### 2.2 Setting

Recruitment occurred online from April to June 2023, targeting people aged 15–29 years from Victoria, Australia. Victoria had a unique experience during the COVID‐19 pandemic, with multiple long and strict lockdowns [13]. There was minimal community transmission until the Omicron wave in January 2022 [14]. By then, most of the population had already been vaccinated against COVID‐19 [15]. At the time of data collection, COVID‐19 was still circulating in the community [14].

### 2.3 Participants

Individuals were eligible to participate in the survey if they were aged 15–29 years and provided informed consent. Participants were excluded if they did not report the outcome variables (history of COVID‐19 and long COVID) or gave inconsistent responses to these variables (e.g., stating no history of COVID‐19, however, currently experiencing long COVID). Although all participants were included in the initial sample analysis, only those with a history of COVID‐19 were included in the subsequent analyses that specifically explored long COVID.

Individuals were recruited online through paid advertisements on Facebook, Instagram, in online community groups, and in the researchers’ networks. COVID‐19 was not mentioned in the advertisements. Participants who completed the survey entered a draw to win A$250.

### 2.4 Variables and Measurement

#### 2.4.1 Primary Outcome

We provided participants with a definition of long COVID, defined as experiencing new health problems that have persisted for more than 1 month after being infected with COVID‐19. Participants reported whether they had ever experienced long COVID, with options “No,” “Yes—and I have been diagnosed by a health professional,” “Yes—I think I have long COVID, but I have not been diagnosed by a health professional,” or “I don't wish to say.” For the primary outcome, we dichotomized the long COVID variable into yes or no (with “yes” combining participants who believed they had long COVID and those who had been diagnosed with long COVID).

#### 2.4.2 Secondary Outcomes

All participants were asked the number of experienced COVID‐19 infections (treated as a continuous variable). For analysis, participants with a history of COVID‐19, but without long COVID symptoms, were labeled “short COVID.”

Participants reporting long COVID answered four questions about the impact of COVID‐19 on their lives. Participants reporting any COVID‐19 infection were asked to rate their current health compared to their health prior to their first COVID‐19 infection on a five‐point scale, ranging from “much worse” to “much better.” These questions originated from the 36‐Item Short Form Survey Instrument (SF36) and were adapted to ask specifically about long COVID [16].

#### 2.4.3 Explanatory Variables

Participants reported the number of COVID‐19 vaccine doses received (treated as a continuous variable). Participants were asked to scale their concern of getting COVID‐19 and long COVID from 0 (no concern) to 10 (extreme concern). All participants were asked about their number of family or friends with a history of long COVID, and how many of these individuals required the participant's support to manage their long COVID. For analysis purposes, this variable was condensed from its initial five categories into three (none/one or two/three or more).

Sociodemographic factors included the participant's age, area of residency in Victoria (dichotomized as either city or regional, based on the reported postal code or region of residency), sex, gender identity, country of birth, Aboriginal and/or Torres Strait Islander ancestry, sexual identity, relationship status, current study status, highest level of education, discretionary income, religiosity, and health service use in past 12 months.

Behavioral characteristics included lifetime alcohol consumption, current smoking status (both e‐cigarettes and other tobacco products), and lifetime recreational drug use. We assessed mental well‐being using the Short Warwick–Edinburgh Mental Well‐Being Scale (SWEMWBS) [17]. Scores could range from 7 to 35 with higher scores indicating better mental well‐being. Existent mental health condition was reported (e.g., anxiety, depression, or schizophrenia), either self‐determined or diagnosed by a health professional.

### 2.5 Statistical Methods

Summary statistics described the sample's demographic and behavioral characteristics, as well as its characteristics related to COVID‐19 and long COVID. Participants were classified into three groups: no reported COVID‐19, short COVID, or long COVID.

The relationship between the primary outcome (long COVID vs. short COVID) and other variables was estimated using logistic regression. Bivariable logistic regression analyses were first conducted with each explanatory variable (Table S2). Variables with a *p* value less than 0.1 in the bivariable logistic regression models or those previously associated with long COVID in the literature were included in the multivariable logistic regression model. Stepwise modeling offered an efficient approach to select the key variables, particularly in this context with a large number of potential predictors [18]. The explanatory variable “Aboriginal and/or Torres Strait Islander ancestry” was dropped from the multivariable model as such a small sample size (*n* = 9) might not provide reliable statistical insights. The explanatory variable “number of affected family or friends that needed participant's support” was also dropped as it was highly correlated with “number of family or friends with long COVID.” Participants with missing data were excluded from the logistic regression models. Stata version 17.0 [19] was used for the data analyses.

### 2.6 Sensitivity Analysis

We conducted a sensitivity analysis to understand the impact of using the NICE definition of long COVID (persisting for ≥4 weeks) [1] over the WHO definition (persisting for ≥12 weeks) [2]. In this analysis, participants reporting symptoms for fewer than 3 months were not considered to have had long COVID. An alternative prevalence was calculated, and factors associated with long COVID with this alternative definition were determined.

## 3 Results

### 3.1 Sample Characteristics

From 1555 people who clicked the survey link, 784 (50.8%) participants provided consent and completed the survey. Of these, 19 records were excluded due to not meeting the age criteria (*n* = 9), no reported outcome variables (*n* = 9), and inconsistency in responses (*n* = 1). The final analysis included 765 young people aged 15–29 years. The sample's demographic and behavioral characteristics are summarized in Table 1. Table S3 shows the sample characteristics categorized by group (no reported COVID‐19, short COVID, and long COVID).

**TABLE 1:** Demographic and behavioral characteristics (*n *= 765).

Variable (*n *= 765)	
Age (years), mean (SD)	23.3 (4.1)
Area of residence, % (*n*)	
City	79.7 (610)
Regional	15.2 (116)
Missing	5.1 (39)
Sex, % (*n*)	
Male	32.4 (248)
Female	66.3 (507)
Missing	1.3 (10)
Gender identity, % (*n*)	
Man	29.8 (228)
Woman	52.2 (399)
Nonbinary and other	16.9 (129)
Missing	1.2 (9)
Country of birth, % (*n*)	
Australia	86.9 (665)
Other	12.6 (96)
Missing	0.5 (4)
Aboriginal and/or Torres Strait Islander ancestry, % (*n*)	
Yes	2.5 (19)
No	96.6 (739)
Missing	0.9 (7)
Sexual identity, % (*n*)	
Heterosexual	44.8 (343)
Other	54.9 (420)
Missing	0.3 (2)
Relationship status, % (*n*)	
Single	38.5 (294)
Relationship	60.9 (465)
Missing	0.8 (6)
Currently studying, % (*n*)	
Yes	54.3 (415)
No	45.2 (346)
Missing	0.5 (4)
Highest level of education, % (*n*)	
High school	23.3 (178)
Post‐high school	76.1 (582)
Missing	0.7 (5)
Discretionary income, % (*n*)	
< A$120	67.7 (518)
≥ A$120	28.9 (221)
Missing	3.4 (26)
Active member of any religious group, % (*n*)	
Yes	9.2 (70)
No	89.8 (687)
Missing	1.1 (8)
Health service use in past 12 months, % (*n*)	
Yes	83.0 (635)
No	16.7 (128)
Missing	0.3 (2)
Ever consumed alcohol, % (*n*)	
Yes	90.2 (690)
No	9.5 (73)
Missing	0.3 (2)
Currently smoking^a^, % (*n*)	
Yes	22.2 (170)
No	77.5 (593)
Missing	0.3 (2)
Ever used recreational drugs, % (*n*)	
Yes	61.3 (469)
No	38.7 (296)
Existent mental health condition, % (*n*)	
Yes	65.9 (504)
No	32.8 (251)
Missing	1.3 (10)
Mental well‐being^b^, mean (SD)	21.0 (3.9)

*Note:* Percentages may not total 100% due to rounding.

^a^ Smoking included e‐cigarettes and other tobacco products.

^b^ Measured with the Short Warwick–Edinburgh Mental Well‐Being Scale (SWEMWBS [22]), score range [7–35], and higher scores indicating better mental well‐being.

### 3.2 COVID‐19

Of 765 participants, 98.2% (*n* = 751) had received at least one dose of the COVID‐19 vaccine. Of all participants, 79.6% (*n* = 609) reported having had one or multiple COVID‐19 infections (Table 2).

**TABLE 2:** COVID‐19‐related characteristics *(n* = 765).

Variable (*n *= 765)	
Number of received vaccine doses, mean (SD)	2.9 (0.7)
Number of COVID‐19 infections, mean (SD)	1.1 (0.8)
Concern of getting COVID‐19 infection, mean (SD)	3.4 (2.7)
Concern of getting long COVID, mean (SD)	4.9 (3.3)
Number of family or friends with long COVID, % (*n*)	
None	50.5 (386)
One or two	35.7 (273)
Three or more	11.8 (90)
Missing	2.1 (16)
Number of affected family or friends that needed participant's support, % (*n*)	
None	84.3 (645)
One or two	12.9 (99)
Three or more	1.4 (11)
Missing	1.3 (10)
COVID‐19 status % (*n*)	
Never had COVID‐19	20.4 (156)
Short COVID‐19 only	68.4 (523)
Long COVID‐19	11.2 (86)

*Note:* Percentages may not total 100% due to rounding.

### 3.3 Long COVID

The prevalence of long COVID was 11.2% (*n* = 86). At the time of the survey, 5.6% reported currently experiencing long COVID (*n *= 43). Most long COVID cases were self‐reported (89.5%, *n* = 77), whereas only 10.5% (*n* = 9) of participants with long COVID symptoms were diagnosed by a health professional. Participants currently experiencing long COVID symptoms had a mean symptom duration of 10.3 months (SD 5.3), whereas the group that had already recovered from long COVID had symptoms for an average of 4.7 months (SD 3.3). The fraction of participants with long COVID increased with a higher number of COVID‐19 infections (one infection: 47/406; two infections: 27/164; three infections: 9/30; four infections: 2/5; and five or more infections: 1/4).

### 3.4 Impact of Long COVID

**FIGURE 1:**
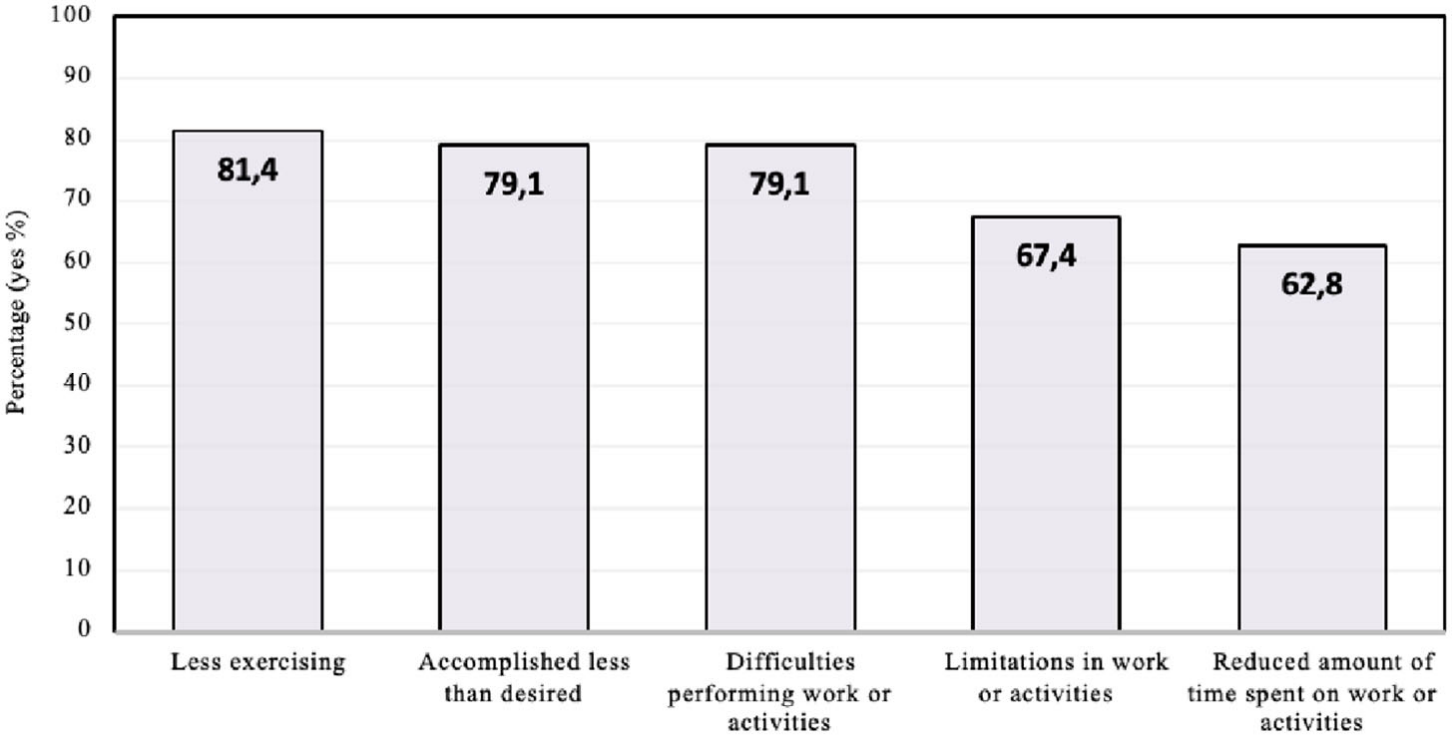
Impact of long COVID on group with current symptoms (*n* = 45). Participants reported the impact during the four weeks prior to the survey.

**FIGURE 2:**
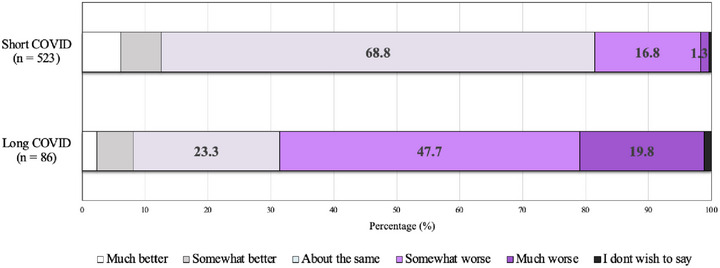
Rating of current health compared to health prior to the first COVID‐19 infection.

Figure 1 shows the impact of long COVID on participants’ lives. Most participants with short COVID reported that their current health was about the same as before their first COVID‐19 infection. Conversely, most participants with long COVID reported a worsened general health (Figure 2).

### 3.5 Associations With Long COVID

Long COVID was negatively associated with older age (Table 3). Participants with long COVID showed greater concern of getting long COVID compared to participants with short. Participants with long COVID had significantly more family or friends with long COVID than participants with short COVID. Participants with long COVID had significantly higher odds of reporting worse general health compared to participants with short COVID. No other variables were significantly associated with long COVID.

**TABLE 3:** Variables associated with long COVID compared to short COVID (bivariable and multivariable logistic regression analysis, 95% confidence intervals [CIs]).

Variable	Short COVID (*n* = 488)	Long COVID (*n* = 75)	OR^a^	95% CI	aOR^b^	95% CI
Age (years), mean (SD)	23.5 (4.2)	23.0 (4.0)	0.97	0.92–1.03	0.92	0.85–0.995
Gender identity, % (*n*)						
Man	30.5 (149)	29.3 (22)	1.00	—	1.00	—
Woman	54.7 (267)	44.0 (33)	0.84	0.47–1.49	0.53	0.27–1.06
Nonbinary and other	14.8 (72)	26.7 (20)	1.88	0.96–3.67	0.78	0.32–1.88
Discretionary income, % (*n*)						
<A$120	68.4 (334)	73.3 (55)	1.00	—	1.00	—
≥A$120	31.6 (154)	26.7 (20)	0.79	0.46–1.36	0.85	0.43–1.69
Currently smoking^c^, % (*n*)						
Yes	76.8 (375)	68.0 (51)	1.56	0.92–2.65	1.33	0.68–2.57
No	23.2 (113)	32.0 (24)	1.00	—	1.00	—
Ever used recreational drugs, % (*n*)						
Yes	99.2 (484)	97.3 (73)	3.32	0.60–18.42	1.99	0.23–16.82
No	0.8 (4)	2.7 (2)	1.00	—	1.00	—
Mental well‐being^d^, mean (SD)	21.2 (3.7)	20.7 (4.5)	0.97	0.91–1.03	1.05	0.97–1.13
Number of received COVID‐19 vaccine doses, mean (SD)	2.8 (0.7)	3.0 (0.8)	1.37	0.96–1.96	1.19	0.77–1.84
Number of COVID‐19 infections, mean (SD)	1.4 (0.7)	1.7 (0.9)	1.55	1.16–2.09	1.30	0.91–1.86
Concern of getting long COVID, mean (SD)	4.6 (3.2)	6.8 (2.7)	1.26	1.16–1.38	1.20	1.09–1.33
Number of family or with long COVID, % (*n*)						
None	54.5 (266)	24.0 (18)	1.00	—	1.00	—
One or two	36.1 (176)	46.7 (35)	2.94	1.61–5.35	2.07	1.06–4.01
Three or more	9.4 (46)	29.3 (22)	7.07	3.52–14.19	4.43	1.92–10.19
Current health compared to prior to COVID‐19, % (*n*)						
Same	67.9 (332)	24.0 (18)	1.00	—	1.00	—
Better	13.1 (64)	8.0 (6)	1.72	0.66–4.51	1.30	0.46–3.62
Worse	19.0 (93)	68.0 (51)	10.08	5.62–18.09	8.22	4.34–15.56

*Note:* The analyses only included participants with complete data on all the variables of interest (any participants missing data were excluded).

Abbreviations: aOR, adjusted odds ratio; OR, odds ratio.

^a^ OR calculated using bivariable logistic regression, comparing long COVID to short COVID (reference group).

^b^ aOR calculated using multivariable logistic regression, comparing long COVID to short COVID (reference group).

^c^ Smoking included e‐cigarettes and other tobacco products.

^d^ Measured with the Short Warwick–Edinburgh Mental Well‐Being Scale (SWEMWBS [22]), score range [7–35], and higher scores indicating better mental well‐being.

### 3.6 Sensitivity Analysis

With a definition of long COVID as persisting for 3 months or more, the prevalence of long COVID in the sample was 9.8% (*n* = 75). Factors associated with long COVID using this definition are shown in Table S4. These factors were consistent with the original analysis, except for age that was no longer associated with long COVID (adjusted odds ratio [aOR]: 0.93; 95% confidence interval [CI]: 0.86–1.01).

## 4 Discussion

### 4.1 Prevalence of Long COVID

This is one of the first studies to measure the prevalence, impacts, and associated factors of long COVID among young people in Victoria, Australia. Of 765 participants, 11% reported they had experienced long COVID, with substantial impacts on their daily life and general health. Most young people who reported experiencing long COVID had not been diagnosed by a healthcare professional and had experienced symptoms for an average of 10 months.

Our study's prevalence of 11% aligns with the prevalence of 14% observed in a survey of 3510 Australian adults as of August 2022 [3]. Both studies defined long COVID as COVID‐19 symptoms persisting for more than 4 weeks. Compared to international data, our study's prevalence is higher than findings from a large‐scale US survey that reported a 5.4% prevalence in young people aged 18–29 years as of October 2023 [4], as well as the prevalence of 1.4% found in the UK among young people aged 17–24 years as of March 2023 [5]. However, both these studies used a definition of long COVID as persisting for ≥12 weeks, compared to our study that used a 4‐week cutoff. They also only included those with a formal diagnosis of long COVID. Both of these criteria would result in a higher prevalence observed in our study.

### 4.2 Impact of Long COVID

Our results suggest that long COVID had considerable impact on young people's daily lives, for example, through difficulties in performing work and exercising. Other studies have documented similar impacts, with one Australian study finding that 86% of adults with long COVID reported a reduced ability to carry out daily activities [3]. Unlike previous research [20], our study found no association between long COVID and mental well‐being. This may be due to the high proportion of participants (66%) reporting a mental health condition. The discrepancy with other research could also be attributed to our use of the SWEMWBS [17], a scale that primarily measures positive mental health. A positive focus in research shifts the narrative from solely treating disorders to promoting mental strengths and emphasizing resilience. This approach is crucial, but it might overlook the negative mental health effects of long COVID.

### 4.3 Factors Associated With Long COVID

Our study unexpectedly found that younger age was associated with long COVID. This finding differs from other research that suggests an inverted U‐shaped relationship between age and long COVID prevalence [21]. Studies indicated an increasing prevalence from childhood [8] through each decade of adult life, followed by a decline around 60 years [21]. However, the relationship was not significant in sensitivity analysis, and it is possible that the finding was due to chance or bias.

Participants with long COVID showed greater concern about getting long COVID than participants with short COVID. This finding may be due to participant's personal experiences with the condition and being more aware of the potential health impacts. Qualitative research suggests that people with long COVID experience unpredictable symptoms [1] and feel uncertain they will ever recover [22, 23]. Research also suggests that long COVID patients face barriers to accessing care, leading to negative impacts on emotional well‐being and recovery [24]. More qualitative research is needed to understand young people's personal experiences with long COVID, access to support services, and the long‐term impacts.

Participants who reported experiencing long COVID had more friends or family members who also had long COVID. This finding may be due to young people seeking support from others experiencing the same condition. Online peer support groups for long COVID were created throughout the pandemic to address gaps in professional care and to advocate for greater awareness of long COVID [25, 26]. Additionally, discussing their long COVID experiences could have prompted others in their social circle to share similar stories. Participants with long COVID could have been more attuned to similar cases, creating the impression of knowing more people with the condition. Network studies could help clarify if the apparent higher prevalence of long COVID within certain social circles is due to increased awareness or actual case clustering.

Gender and sex were not associated with long COVID. Some studies highlighted female sex as a potential risk factor for long COVID in adults [27, 28]. This trend is less evident in adolescent populations [8, 29]. The underlying causation of this gender disparity in long COVID risk remains unclear. Biological (sex) factors might explain why female adults are more at risk for long COVID. For example, females showed a more robust immune activation than males in acute COVID‐19 [30], possibly contributing to the persistence of long COVID symptoms in females. Sociocultural (gender) roles could also play a crucial part. Cisgender women, who more predominantly occupy roles in healthcare and caregiving [31], might face increased viral exposure. Additionally, gender‐based differences in healthcare‐seeking behavior [32] might lead to more frequent reporting of long COVID symptoms among cisgender women. These factors may manifest differently in younger populations, including our study group with an average age of 23. Further research in both biological (sex) and sociocultural (gender) differences regarding long COVID in this age group is required to deepen the understanding of this relationship.

Although not included in multivariable analysis due to small numbers, there was a markedly high proportion of participants who identified as Aboriginal and Torres Strait Islander reporting long COVID (21%, 4/19). This demonstrates a need for focused and community‐led research and management strategies for long COVID among Aboriginal and Torres Strait Islander people.

Finally, COVID‐19 vaccination is a known protective factor against long COVID in children and adults [33, 34, 35]. Our study observed no association, likely because nearly all participants (98%) were vaccinated.

### 4.4 Implications

Our study demonstrates that a significant minority of young people in Victoria reported experiencing long COVID, with many experiencing symptoms for >10 months. A recent review identified there are a small number of services in Australia that provide long COVID rehabilitation and support to patients to manage symptoms [10]. However, many have since been closed [36]. Most services require a referral from a general practitioner or specialist and include support from allied health professionals like physiotherapists and psychologists. Our findings suggest that young people with long COVID will likely also require support from their employers and education institutes to enable flexible work and study arrangements [37].

Most of our participants had not been diagnosed with long COVID by a healthcare professional; however, we do not know if this is because they had not sought diagnosis for the condition, or if they had sought care but not received a formal diagnosis. Further work may be needed to ensure that general practitioners are confident in diagnosing long COVID or providing alternative diagnoses, and that young people with chronic conditions can navigate the healthcare system and access support when needed. Long COVID research shows that many people with the condition feel invalidated, dismissed, or disbelieved when seeking help from medical professionals [38, 39, 40, 41]. This research shows that feeling heard and believed can be as important for well‐being as any medical treatment. Clinicians can support patients by listening to and validating patients’ concerns and sharing their own frustrations with lack of scientific and clinical knowledge around long COVID [38, 39]. Long COVID patients should be offered multidisciplinary patient‐centered integrated care and active self‐management [10, 42]. However, more research into treatment and prevention of long COVID is needed [43].

Given its prevalence and impact, work is needed to raise awareness and educate the public about long COVID. A longitudinal cohort study reinforces this by showing that only one‐third of its participants considered the Victorian government's information on long COVID sufficient [44]. Increasing awareness about long COVID could facilitate earlier detection and timely, effective support, while setting realistic expectations and reducing the stigma around long COVID [22].

### 4.5 Limitations and Strengths

Our study has several limitations. First, COVID was self‐reported, which may have led to over‐ or underreporting and misdiagnosis. Diagnosis was based on a single item rather than a validated diagnostic tool or questionnaire. Given that even healthcare professionals find diagnosing long COVID challenging [45], people without medical training might have attributed symptoms of other conditions to long COVID. However, patient‐reported outcomes are central to identification of long COVID, and self‐reported outcomes are used commonly in long COVID research [40, 46]. Second, our study was cross‐sectional, and we cannot establish a temporal relationship between variables. We are also unable to verify onset dates and duration of symptoms. Implementing a national, longitudinal screening program for long COVID in Australia, modeled after successful international initiatives [4, 5], is recommended to follow its progression over time. Third, our study used convenience sampling; therefore, it is unlikely participants represent the general population of young people in Australia. Recruiting participants online means we likely did not reach participants with lower levels of education and digital literacy. Studies have shown that those with greater digital health literacy are more likely to take precautions to prevent COVID infection, which may have affected our reported prevalence rates [47, 48]. A strength of the study was that it was not advertised as a COVID‐19 study, so selection bias towards those with strong feelings about long COVID was likely minimized. However, the advertising of the survey topic as “Sex, Drugs, and Rock‘n'Roll” means there may have been a bias towards volunteers of particular social and cultural groups, for example, we have a high number of participants identifying as LGBTQIA+. Although there is no evidence that LGBTQIA+ young people have disproportionate rates of long COVID, we have previously shown that this group experienced greater psychological distress during the pandemic [49].

## 5 Conclusions

The unique perspective of this study, from young people, as opposed to clinicians, contributes to the existing literature. Our study suggests that long COVID in young people is a significant public health concern, affecting 11% of our sample. Most reported negative impacts on their health and daily lives. Young people with long COVID were concerned about getting the condition again and often had others in their social circle who also experienced long COVID. General practitioners should be aware of the high burden of suspected long COVID in young people and consider potential supports to mitigate its long‐term effects on the health and well‐being of this population.

## Author Contributions

Study design: CHD, AO, SE, ST, MSCL. Data collection: AO, SE, MSCL. Methodology: RE, CHD, ALW, AA, ST, MSCL. Data analysis: RE, AO, SE, ALW. Writing: RE, MSCL. Writing and editing: all authors.

## Ethics Statement

Ethical approval was given by the Alfred Health Research Ethics Committee (project number 326/08).

## Consent

All participants gave informed consent.

## Conflicts of Interest

The authors declare no conflicts of interest.

## Supporting information




**Supporting Table 1:** COVID‐19‐related questions from Sex, Drugs, and Rock‘n’Roll (SDRR) study 2023.
**Supporting Table 2:** Bivariable analysis of each explanatory variable (bivariable logistic regression analysis, 95% confidence intervals).
**Supporting Table 3:** Sample characteristics categorized by group (no reported COVID‐19, short COVID and long COVID).
**Supporting Table 4:** Sensitivity analysis using symptoms persisting for 3 or more months as the definition of long COVID. Variables associated with long COVID compared to short COVID (bivariable and multivariable logistic regression analysis, 95% confidence intervals).

## Data Availability

The data that support this study cannot be publicly shared due to ethical or privacy reasons and may be shared upon reasonable request to the corresponding author if appropriate.
