# Supplementary material for: Understanding Long COVID Among Young People in Victoria, Australia: Prevalence, Impact, and Associated Factors
Source: Public Health Chall. 2025 Oct 21;4(4):e70144. doi: 10.1002/puh2.70144 (PMC12539282; doi:10.1002/puh2.70144)
Supplement: Supplementary file 1 — Supporting Table 1: COVID‐19‐related questions from Sex, Drugs, and Rock‘n’Roll (SDRR) study 2023. Supporting Table 2: Bivariable analysis of each explanatory variable (bivariable logistic regression analysis, 95% confidence intervals). Supporting Table 3: Sample characteristics categorized by group (no reported COVID‐19, short COVID and long COVID). Supporting Table 4: Sensitivity analysis using symptoms persisting for 3 or more months as the definition of long COVID. Variables associated with long COVID compared to short COVID (bivariable and multivariable logistic regression analysis, 95% confidence intervals). [file PUH2-4-e70144-s001.docx]

**Supplementary Table S1:** COVID-19-related questions from Sex, Drugs, and Rock'n'Roll (SDRR) study 2023

**Table S1**COVID-19-related questions from the Sex, Drugs, and Rock'n'Roll (SDRR) study 2023

| Number | Question | Options |
| --- | --- | --- |
| 1 | How many doses of the COVID-19 vaccine have you received? | 0 doses (I am not vaccinated); 1 dose; 2 doses; 3 doses; 4 doses; 5 or more doses; Unsure/Don’t know; I don’t wish to say. |
| 2 | How many times have you had COVID-19 since the start of the pandemic (December 2019)? *Had COVID-19 means tested positive by PCR or rapid antigen test (RAT) or both, with at least one month since last testing positive.* | I have never had COVID-19; Once; Twice; Three times; Four times; Five times or more; I don’t wish to say. |
| 3  (if question 2 $\geq$ 0) | Compared to before your first COVID-19 infection, how would you rate your health in general now? | Much better now; Somewhat better now; About the same as before COVID-19 infection; Somewhat worse now; Much worse now; I don’t wish to say. |
| 4 | How concerned are you about getting COVID? *Rate between 0–10, where 0 is not at all concerned and 10 is extremely concerned.*   1. Short term (acute) covid infection; and 2. Long term (long covid) infection. | [Numeric answer, minimum 0, maximum 10]; I don’t wish to say. |
| 5^a^ | To what extent do you agree or disagree with the following statements about long COVID?   1. The risk of long COVID motivates me to wear a face mask in indoor public settings; 2. The risk of long COVID motivates me to get vaccinated/stay up to date with vaccines; 3. The risk of long COVID motivates me to work and/or study from home as much as possible; 4. The risk of long COVID motivates me to avoid attending small social gatherings (i.e., family dinners, holiday parties); 5. The risk of long COVID motivates me to avoid large social gatherings (i.e. concerts, large weddings); 6. To prevent long COVID it would be acceptable for the Victorian Government to require wearing a face mask in indoor public settings; and 7. The government is providing enough information about long COVID and the risks associated with having long COVID. | Totally agree; Somewhat agree; Neutral; Somewhat disagree; Totally disagree; Don’t know/Not sure. |
| 6 | Have you ever experienced long COVID? | No; Yes, and I have been diagnosed with long COVID by a health professional; Yes, I think I have long COVID but I have not been diagnosed by a health professional; I don’t wish to say. |
| 7  (if question 6 = yes) | Do you still have long COVID? | Yes, I am still experiencing symptoms; No, all my symptoms have completely resolved; Prefer not to say. |
| 8  (if question 7 = yes) | How long do you think you have had long COVID for? | [Numeric answer, minimum 1, maximum 36] months. |
| 9  (if question 7 = no) | How long do you think you had long COVID for? | [Numeric answer, minimum 1, maximum 36] months. |
| 10 | During the past four weeks, as a result of your long COVID, have you had any of the following problems with your work or other regular daily activities?   1. Cut down the amount of time you spent on work or other activities; 2. Accomplished less than you would like; 3. Were limited in the kind of work or other activities; 4. Had difficulty performing the work or other activities (for example, it took extra effort); and 5. Couldn’t exercise as much as before. | Yes; No; I don’t wish to say. |
| 11 | How many of your family or friends have experienced long COVID? | None; 1 or 2; 3 or 4; Five to ten; More than ten; Prefer not to say. |
| 12 | How many of your family or friends have required your support to help them manage long COVID? | None; 1 or 2; 3 or 4; Five to ten; More than ten; Prefer not to say. |

^a^ Question 5 was not analyzed in this paper.

**Supplementary Table S2:** Bivariable analysis of each explanatory variable (bivariable logistic regression analysis, 95% confidence intervals)

**Table S2**
Bivariable analysis of each explanatory variable (bivariable logistic regression analysis, 95% confidence intervals)

| Variable | *n* | OR^a^ | 95% CI |
| --- | --- | --- | --- |
| Age (years) | S: 523; L: 86 | 0.98 | 0.93–1.04 |
| Area of residence | S: 497; L: 85 |  |  |
| City |  | 0.97 | 0.51–1.84 |
| Regional |  | 1.00 | - |
| Sex | S: 518; L: 84 |  |  |
| Male |  | 1.00 | - |
| Female |  | 1.06 | 0.65–1.75 |
| Gender identity | S: 519; L: 85 |  |  |
| Man |  | 1.00 | - |
| Woman |  | 0.89 | 0.52–1.52 |
| Non-binary and other |  | 1.60 | 0.84–3.03 |
| Country of birth | S: 519; L: 86 |  |  |
| Australia |  | 1.00 | - |
| Other |  | 0.89 | 0.44–1.80 |
| Aboriginal and/or Torres Strait Islander ancestry | S: 519; L: 85 |  |  |
| Yes |  | **3.61** | 1.03–12.62 |
| No |  | 1.00 | - |
| Sexual identity | S: 522; L: 86 |  |  |
| Heterosexual |  | 1.00 | - |
| Other |  | 1.12 | 0.71–1.77 |
| Relationship status | S: 520; L: 86 |  |  |
| Single |  | 1.00 | - |
| Relationship |  | 1.48 | 0.90–2.42 |
| Currently studying | S: 521; L: 85 |  |  |
| Yes |  | 0.85 | 0.54–1.34 |
| No |  | 1.00 | - |
| Highest level of education | S: 520; L: 85 |  |  |
| High school |  | 1.00 | - |
| Post-high school |  | 0.69 | 0.41–1.15 |
| Discretionary income | S: 504; L: 84 |  |  |
| < A$120 |  | 1.00 | - |
| ≥ A$120 |  | 0.80 | 0.47–1.35 |
| Active member of any religious group | S: 520; L: 85 |  |  |
| Yes |  | 0.92 | 0.40–2.12 |
| No |  | 1.00 | - |
| Health service use in past 12 month | S: 521; L: 86 |  |  |
| Yes |  | 1.67 | 0.83–3.36 |
| No |  | 1.00 | - |
| Ever consumed alcohol | S: 522; L: 86 |  |  |
| Yes |  | 0.99 | 0.45–2.17 |
| No |  | 1.00 | - |
| Currently smoking^b^ | S: 521; L: 86 |  |  |
| Yes |  | **1.55** | 0.94–2.55 |
| No |  | 1.00 | - |
| Ever used recreational drugs | S: 523; L: 86 |  |  |
| Yes |  | **3.74** | 0.88–16.00 |
| No |  | 1.00 | - |
| Existent mental health condition | S: 516; L: 85 |  |  |
| Yes |  | 1.22 | 0.74–2.02 |
| No |  | 1.00 | - |
| Mental well-being^c^ | S: 522; L: 84 | 0.96 | 0.90–1.02 |
| Number of received vaccine doses | S: 520; L: 85 | **1.40** | 1.00–1.97 |
| Number of COVID-19 infections | S: 523; L: 86 | **1.57** | 1.18–2.09 |
| Concern of getting COVID-19 infection | S: 522; L: 86 | **1.24** | 1.14–1.34 |
| Concern of getting long COVID | S: 522; L: 86 | **1.21** | 1.11–1.32 |
| Number of family or friends with long COVID | S: 512; L: 81 |  |  |
| None |  | 1.00 | - |
| One or two |  | **2.80** | 1.59–4.94 |
| Three or more |  | **6.81** | 3.49–13.29 |
| Number of affected family or friends that needed participant’s support | S: 516; L: 83 |  |  |
| None |  | 1.00 | - |
| One or two |  | **1.73** | 0.94–3.17 |
| Three or more |  | **4.61** | 0.76–28.08 |
| Current health compared to prior to COVID-19 | S: 521; L: 85 |  |  |
| Same |  | 1.00 | - |
| Better |  | 1.91 | 0.78–4.69 |
| Worse |  | **10.99** | 6.30–19.17 |

Bold represents statistically significant at *p* < 0.1.
CI = confidence interval; L = long COVID group; OR = odds ratio; S = short COVID group.
 ^a^ OR calculated using bivariable logistic regression, comparing long COVID to short COVID (reference group).

^b^ Smoking included e-cigarettes and other tobacco products. ^c^ Measured with the Short Warwick-Edinburgh Mental Well-being Scale (SWEMWBS (23)), score range [7–35], higher scores indicating higher mental well-being.
Note: Each bivariable logistic regression analysis only included participants with complete data on that specific variable.

**Supplementary Table S3:** Sample characteristics categorized by group (no reported COVID-19, short COVID and long COVID)


**Table S3**Sample characteristics categorized by group (no reported COVID-19, short COVID and long COVID)

| Variable | All participants^a^ (*n* = 765*)* | No reported COVID-19^a^ (*n* = 156) | Short COVID^a^ (*n* = 523) | Long COVID^a^ (*n* = 86) |
| --- | --- | --- | --- | --- |
| Age (years), mean (SD) | 23.3 (4.1) | 23.1 (4.0) | 23.3 (4.2) | 23.0 (3.9) |
| Area of residence, % (n) |  |  |  |  |
| City | 79.7 (610) | 73.7 (115) | 80.9 (423) | 83.7 (72) |
| Regional | 15.2 (116) | 18.6 (29) | 14.2 (74) | 15.1 (13) |
| Missing | 5.1 (39) | 7.7 (12) | 5.0 (26) | 1.2 (1) |
| Sex, % (n) |  |  |  |  |
| Male | 32.4 (248) | 35.3 (55) | 31.9 (167) | 30.2 (26) |
| Female | 66.3 (507) | 62.8 (98) | 67.1 (351) | 67.4 (58) |
| Missing | 1.3 (10) | 1.9 (3) | 1.0 (5) | 2.3 (2) |
| Gender identity, % (n) |  |  |  |  |
| Man | 29.8 (228) | 28.9 (45) | 30.2 (158) | 29.1 (25) |
| Woman | 52.2 (399) | 52.6 (82) | 53.2 (278) | 45.4 (39) |
| Non-binary and other | 16.9 (129) | 16.0 (25) | 15.9 (83) | 24.4 (21) |
| Missing | 1.2 (9) | 2.6 (4) | 0.8 (4) | 1.2 (1) |
| Country of birth, % (n) |  |  |  |  |
| Australia | 86.9 (665) | 87.8 (137) | 86.4 (452) | 88.4 (76) |
| Other | 12.6 (96) | 12.2 (19) | 12.8 (67) | 11.6 (10) |
| Missing | 0.5 (4) | - | 0.8 (4) | - |
| Aboriginal and/or Torres Street Islander ancestry, % (n) |  |  |  |  |
| Yes | 2.5 (19) | 5.1 (8) | 1.3 (7) | 4.7 (4) |
| No | 96.6 (739) | 93.6 (146) | 97.9 (512) | 94.2 (81) |
| Missing | 0.9 (7) | 1.3 (2) | 0.8 (4) | 1.2 (1) |
| Sexual identity, % (n) |  |  |  |  |
| Heterosexual | 44.8 (343) | 43.0 (67) | 45.7 (239) | 43.0 (37) |
| Other | 54.9 (420) | 56.4 (88) | 54.1 (283) | 57.0 (49) |
| Missing | 0.3 (2) | 0.6 (1) | 0.2 (1) | - |
| Relationship status, % (n) |  |  |  |  |
| Single | 38.5 (294) | 41.7 (65) | 38.8 (203) | 30.2 (26) |
| Relationship | 60.9 (465) | 56.4 (88) | 60.6 (317) | 69.8 (60) |
| Missing | 0.8 (6) | 1.9 (3) | 0.6 (3) | - |
| Currently studying, % (n) |  |  |  |  |
| Yes | 54.3 (415) | 55.8 (87) | 54.5 (285) | 50.0 (43) |
| No | 45.2 (346) | 43.6 (68) | 45.1 (236) | 48.8 (42) |
| Missing | 0.5 (4) | 0.6 (1) | 0.4 (2) | 1.2 (1) |
| Highest level of education, % (n) |  |  |  |  |
| High school | 23.3 (178) | 23.7 (37) | 22.2 (116) | 29.1 (25) |
| Post-high school | 76.1 (582) | 75.6 (118) | 77.3 (404) | 69.8 (60) |
| Missing | 0.7 (5) | 0.6 (1) | 0.6 (3) | 1.2 (1) |
| Discretionary income, % (n) |  |  |  |  |
| < A$120 | 67.7 (518) | 68.6 (107) | 66.7 (349) | 72.1 (62) |
| ≥ A$120 | 28.9 (221) | 28.2 (44) | 29.6 (155) | 25.6 (22) |
| Missing | 3.4 (26) | 3.2 (5) | 3.6 (19) | 2.3 (2) |
| Active member of any religious group, % (n) |  |  |  |  |
| Yes | 9.2 (70) | 10.9 (17) | 8.8 (46) | 8.1 (7) |
| No | 89.8 (687) | 86.5 (135) | 90.6 (474) | 90.7 (78) |
| Missing | 1.1 (8) | 2.6 (4) | 0.6 (3) | 1.2 (1) |
| Health service use in past 12 months, % (n) |  |  |  |  |
| Yes | 83.0 (635) | 84.6 (132) | 81.6 (427) | 88.4 (76) |
| No | 16.7 (128) | 15.4 (24) | 18.0 (94) | 11.6 (10) |
| Missing | 0.3 (2) | - | 0.4 (2) | - |
| Ever consumed alcohol, % (n) |  |  |  |  |
| Yes | 90.2 (690) | 88.5 (138) | 90.6 (474) | 90.7 (78) |
| No | 9.5 (73) | 10.9 (17) | 9.2 (48) | 9.3 (8) |
| Missing | 0.3 (2) | 0.6 (1) | 0.2 (1) | - |
| Currently smoking^a^, % (n) |  |  |  |  |
| Yes | 22.2 (170) | 15.4 (24) | 22.8 (119) | 31.4 (27) |
| No | 77.5 (593) | 84.6 (132) | 76.9 (402) | 68.6 (59) |
| Missing | 0.3 (2) | - | 0.4 (2) | - |
| Ever used recreational drugs, % (n) |  |  |  |  |
| Yes | 61.3 (469) | 54.5 (85) | 62.7 (328) | 65.1 (56) |
| No | 38.7 (296) | 45.4 (71) | 37.3 (195) | 34.9 (30) |
| Existent mental health condition, % (n) |  |  |  |  |
| Yes | 65.9 (504) | 65.4 (102) | 65.4 (342) | 69.8 (60) |
| No | 32.8 (251) | 33.3 (52) | 33.3 (174) | 29.1 (25) |
| Missing | 1.3 (10) | 1.3 (2) | 1.3 (7) | 1.2 (1) |
| Mental well-being^b^, mean (SD) | 21.0 (3.9) | 20.9 (3.8) | 21.1 (3.7) | 20.4 (4.5) |
| Number of received vaccine doses, mean (SD) | 2.9 (0.7) | 3.0 (0.7) | 2.8 (0.7) | 3.0 (0.8) |
| Number of COVID-19 infections, mean (SD) | 1.1 (0.8) | - | 1.4 (0.6) | 1.6 (0.9) |
| Concern of getting COVID-19 infection, mean (SD) | 3.4 (2.7) | 3.4 (2.8) | 3.2 (2.6) | 4.7 (2.8) |
| Concern of getting long COVID, mean (SD) | 4.9 (3.3) | 4.8 (3.5) | 4.6 (3.2) | 6.6 (2.8) |
| Number of family or friends with long COVID, % (n) |  |  |  |  |
| None | 50.5 (386) | 50.6 (79) | 54.7 (286) | 24.4 (21) |
| One or two | 35.7 (273) | 35.9 (56) | 34.4 (180) | 43.0 (37) |
| Three or more | 11.8 (90) | 13.5 (21) | 8.8 (46) | 26.7 (23) |
| Missing | 2.1 (16) | - | 2.1 (11) | 5.8 (5) |
| Number of affected family or friends that needed participant’s support, % (n) |  |  |  |  |
| None | 84.3 (645) | 84.0 (131) | 85.9 (449) | 75.6 (65) |
| One or two | 12.9 (99) | 12.2 (19) | 12.2 (64) | 18.6 (16) |
| Three or more | 1.4 (11) | 3.9 (6) | 0.6 (3) | 2.3 (2) |
| Missing | 1.3 (10) | - | 1.3 (7) | 3.5 (3) |

^a^ Smoking included e-cigarettes and other tobacco products.
 ^b^ Measured with the Short Warwick-Edinburgh Mental Well-being Scale (SWEMWBS (23)), score range [7–35], higher scores indicating better mental well-being.
Note: Percentages may not total 100% due to rounding.

**Supplementary Table S4**Sensitivity analysis using symptoms persisting for 3 or more months as the definition of long COVID. Variables associated with long COVID compared to short COVID (bivariable and multivariable logistic regression analysis, 95% confidence intervals)

| Variable | Short COVID  (*n* = 496) | Long COVID  (*n* = 67) | OR^a^ | 95% CI | aOR^b^ | 95% CI |
| --- | --- | --- | --- | --- | --- | --- |
| Age (years), mean (SD) | 23.5 (4.2) | 22.9 (3.9) | 0.97 | 0.91–1.03 | 0.93 | 0.86–1.01 |
| Gender identity, % (*n*) |  |  |  |  |  |  |
| Man | 30.9 (153) | 26.9 (18) | 1.00 | - | 1.00 | - |
| Woman | 54.7 (271) | 43.3 (29) | 0.91 | 0.49–1.69 | 0.60 | 0.29–1.26 |
| Non-binary and other | 14.5 (72) | 29.9 (20) | **2.36** | **1.18-4.73** | 1.11 | 0.35–2.77 |
| Discretionary income, % (*n*) |  |  |  |  |  |  |
| < A$120 | 68.4 (339) | 74.6 (50) | 1.00 | - | 1.00 | - |
| ≥ A$120 | 31.6 (157) | 25.4 (17) | 0.73 | 0.41–1.31 | 0.80 | 0.38–1.68 |
| Currently smoking^c^, % (n) |  |  |  |  |  |  |
| Yes | 76.4 (379) | 70.2 (47) | 1.38 | 0.79–2.42 | 1.12 | 0.55–2.27 |
| No | 23.6 (117) | 29.8 (20) | 1.00 | - | 1.00 | - |
| Ever used recreational drugs, % (n) |  |  |  |  |  |  |
| Yes | 99.2 (492) | 97.3 (65) | 3.78 | 0.68–21.1 | 2.35 | 0.29–18.78 |
| No | 0.8 (4) | 2.7 (2) | 1.00 | - | 1.00 | - |
| Mental well-being^d^, mean (SD) | 21.1 (3.8) | 20.9 (4.5) | 0.98 | 0.92–1.05 | 1.07 | 0.99–1.16 |
| Number of received COVID-19 vaccine doses, mean (SD) | 2.8 (0.7) | 3.0 (0.8) | 1.31 | 0.90–1.90 | 1.10 | 0.70–1.73 |
| Number of COVID-19 infections, mean (SD) | 1.4 (0.7) | 1.7 (0.9) | **1.58** | **1.17–2.15** | 1.37 | 0.94–1.99 |
| Concern of getting long COVID, mean (SD) | 4.7 (3.2) | 6.9 (2.7) | **1.28** | **1.17–1.41** | **1.21** | **1.09–1.35** |
| Number of family or with long COVID, % (*n*) |  |  |  |  |  |  |
| None | 53.8 (267) | 25.4 (17) | 1.00 | - | 1.00 | - |
| One or two | 36.5 (181) | 44.8 (30) | **2.60** | **1.39–4.86** | 1.77 | 0.88-3.56 |
| Three or more | 9.7 (48) | 29.9 (20) | **6.54** | **3.20–13.39** | **3.99** | **1.67–9.51** |
| Current health compared to prior to COVID-19, % (*n*) |  |  |  |  |  |  |
| Same | 67.5 (335) | 20.9 (14) | 1.00 | - | 1.00 | - |
| Better | 12.9 (64) | 9.0 (6) | 2.24 | 0.83–6.06 | 1.68 | 0.59–4.84 |
| Worse | 19.6 (97) | 70.2 (47) | **11.60** | **6.12-21.95** | **9.23** | **4.62–18.42** |

Bold represents statistically significant at *p* < 0.05.
aOR = adjusted odds ratio; CI = confidence interval; OR = odds ratio.
 ^a^ OR calculated using bivariable logistic regression, comparing long COVID to short COVID (reference group).

^b^ aOR calculated using multivariable logistic regression, comparing long COVID to short COVID (reference group).
 ^c^ Smoking included e-cigarettes and other tobacco products. ^d^ Measured with the Short Warwick-Edinburgh Mental Well-being Scale (SWEMWBS (22)), score range [7–35], higher scores indicating better mental well-being.
Note: The analyses only included participants with complete data on all the variables of interest.
